# P-2016. Nirmatrelvir-ritonavir and molnupiravir for the outpatient treatment of Covid-19: a population-based cohort study

**DOI:** 10.1093/ofid/ofae631.2173

**Published:** 2025-01-29

**Authors:** Anselm Jorda, Dominik Ensle, Hubert Eser, Florentin Glötzl, Benjamin Riedl, Ursula Karnthaler, Markus Zeitlinger

**Affiliations:** Medical University of Vienna, Vienna, Wien, Austria; Municipal Department for Public Health Services of the City of Vienna, Vienna, Austria, Vienna, Wien, Austria; Municipal Department for Information Technology of the City of Vienna, Vienna, Austria, Vienna, Wien, Austria; Institute for Ecological Economics, Department for Socioeconomics, Vienna University of Economics and Business, Austria, Vienna, Wien, Austria; Institute for Ecological Economics, Department for Socioeconomics, Vienna University of Economics and Business, Austria, Vienna, Wien, Austria; Municipal Department for Public Health Services of the City of Vienna, Vienna, Austria, Vienna, Wien, Austria; Department of Clinical Pharmacology, Vienna, Wien, Austria

## Abstract

**Background:**

The real-world effectiveness of the oral antivirals nirmatrelvir-ritonavir and molnupiravir against the SARS-CoV-2 Omicron variant remains uncertain. Our aim was to evaluate their effectiveness in non-hospitalized adults with Covid-19 in Vienna, Austria.

**Figure 1:**
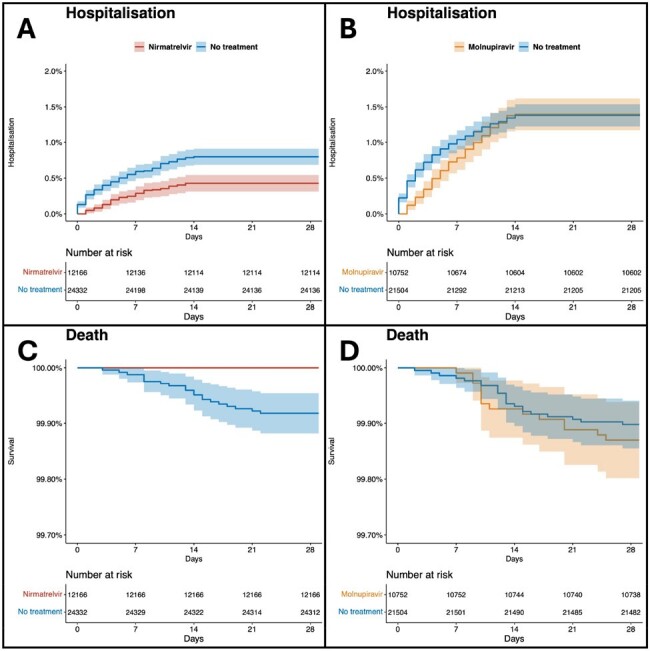
Kaplan-Meier curves of (A) the hospitalization within 28 days between nirmatrelvir-ritonavir vs matched untreated controls, (B) hospitalization within 28 days between molnupiravir vs matched untreated controls, (C) all-cause death within 28 days between nirmatrelvir-ritonavir vs matched untreated controls, and (D) all-cause death within 28 days between molnupiravir vs matched untreated controls. All curves were based on propensity score-matched analysis. The shaded areas indicate 95% confidence intervals. The Y-axis does not range from 0 to 100% because of the low incidence.

**Methods:**

This observational study used data from the Municipal Department for Public Health Services of Vienna to identify non-hospitalized adults with confirmed SARS-CoV-2 infection between Jan-2022 and May-2023. Untreated controls were matched to nirmatrelvir-ritonavir users and molnupiravir users in a 2:1 ratio using propensity scores. Outcomes were hospitalization and all-cause death within 28 days.

**Figure 2:**
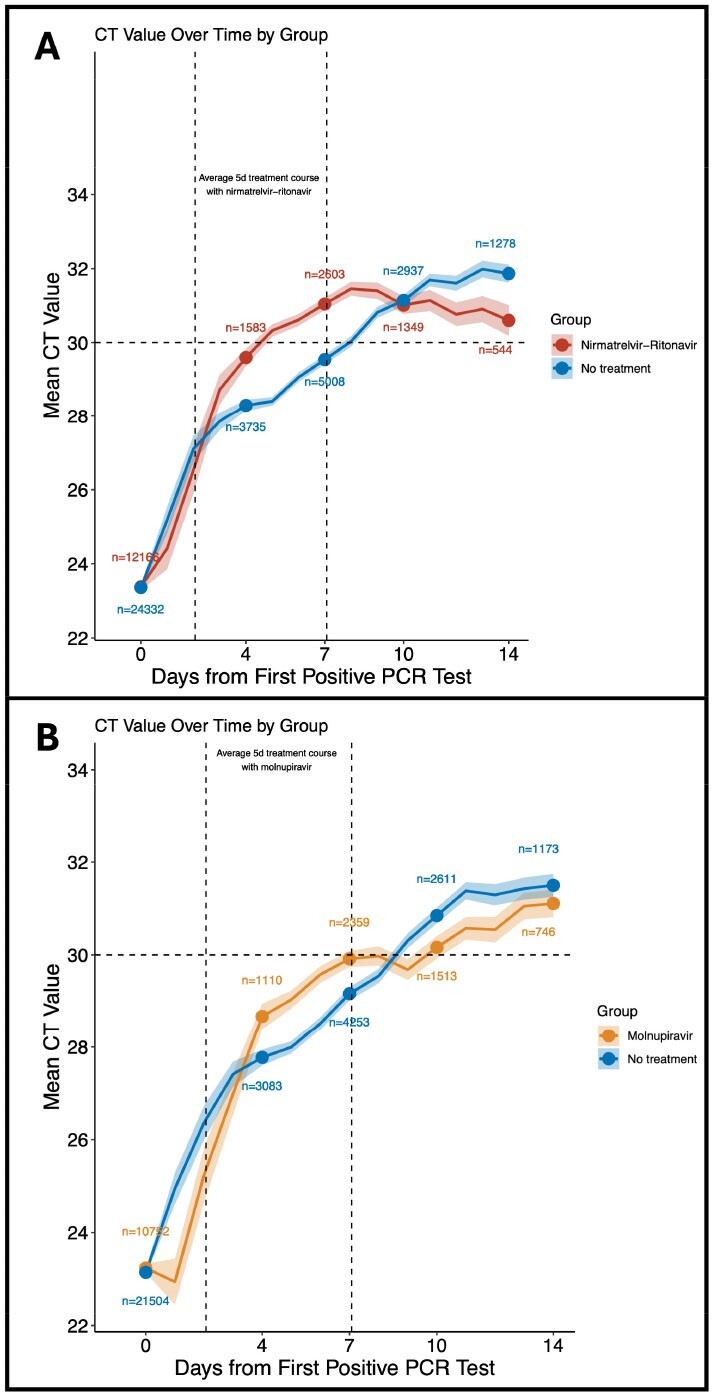
CT values over time between (A) nirmatrelvir-ritonavir vs untreated controls and (B) molnupiravir vs untreated controls.

Shaded areas represent the 95% CI of the mean CT value. Dots indicate the time points of group comparisons using an independent t-test. The numbers indicate the number of subjects per group on days 0, 4, 7, 10, and 14. The results of the mean differences with 95% confidence intervals are provided in Supplementary Tables 4 and 5 (wherever the 95% confidence intervals did not cross, a statistically significant difference was found). The horizontal dotted line indicates a CT value of 30, which is usually defined as the threshold for the end of the infection and contagiousness. Vertical dotted lines indicate the presumed start and end of the 5-day treatment course with (A) nirmatrelvir-ritonavir or (B) molnupiravir.

**Results:**

We identified 113,399 eligible cases with SARS-CoV-2 infection. Of 90,481 untreated controls, 24,332 were matched to 12,166 nirmatrelvir-ritonavir users and 21,504 were matched to 10,752 molnupiravir users. In the nirmatrelvir-ritonavir matched analysis set, 52 (0.43%) of 12166 nirmatrelvir-ritonavir users and 194 (0.8%) of 24332 matched controls were hospitalized (HR 0.53, 95%CI 0.39-0.73). No (0%) nirmatrelvir-ritonavir users and 20 (0.08%) matched controls died (p< 0.0001) (Figure 1). This finding for nirmatrelvir-ritonavir was independent of vaccination status but was restricted to people aged ≥60 years. In the molnupiravir-matched analysis set, 150 (1.4%) of 10752 molnupiravir users and 297 (1.38%) of 21504 matched controls were hospitalized (HR 1.01, 95%CI 0.83-1.23), with 14 (0.13%) and 21 (0.1%) deaths, respectively (HR 1.27, 95%CI 0.65-2.49) (Figure 1). Over 96% of the patients included were vaccinated. For both antiviral agents a rebound in viral load was observed after an initially accelerated viral clearance (Figure 2).

**Conclusion:**

Among outpatients aged ≥60 years with Covid-19 caused by the Omicron variant, treatment with nirmatrelvir-ritonavir was associated with a significantly lower risk of hospitalization and all-cause mortality within 28 days. This finding was not observed in molnupiravir users and younger nirmatrelvir-ritonavir users.

**Disclosures:**

All Authors: No reported disclosures

